# Design and Construction of an Inexpensive Homemade Plant Growth Chamber

**DOI:** 10.1371/journal.pone.0126826

**Published:** 2015-05-12

**Authors:** Fumiaki Katagiri, Dario Canelon-Suarez, Kelsey Griffin, John Petersen, Rachel K. Meyer, Megan Siegle, Keisuke Mase

**Affiliations:** 1 Department of Plant Biology, Microbial and Plant Genomics Institute, University of Minnesota, Saint Paul, Minnesota, United States of America; 2 Department of Computer Science and Engineering, University of Minnesota, Minneapolis, Minnesota, United States of America; Leibniz Institute of Plant Biochemistry, GERMANY

## Abstract

Plant growth chambers produce controlled environments, which are crucial in making reproducible observations in experimental plant biology research. Commercial plant growth chambers can provide precise controls of environmental parameters, such as temperature, humidity, and light cycle, and the capability via complex programming to regulate these environmental parameters. But they are expensive. The high cost of maintaining a controlled growth environment is often a limiting factor when determining experiment size and feasibility. To overcome the limitation of commercial growth chambers, we designed and constructed an inexpensive plant growth chamber with consumer products for a material cost of $2,300. For a comparable growth space, a commercial plant growth chamber could cost $40,000 or more. Our plant growth chamber had outside dimensions of 1.5 m (W) x 1.8 m (D) x 2 m (H), providing a total growth area of 4.5 m^2^ with 40-cm high clearance. The dimensions of the growth area and height can be flexibly changed. Fluorescent lights with large reflectors provided a relatively spatially uniform photosynthetically active radiation intensity of 140–250 μmoles/m^2^/sec. A portable air conditioner provided an ample cooling capacity, and a cooling water mister acted as a powerful humidifier. Temperature, relative humidity, and light cycle inside the chamber were controlled via a z-wave home automation system, which allowed the environmental parameters to be monitored and programmed through the internet. In our setting, the temperature was tightly controlled: 22.2°C±0.8°C. The one-hour average relative humidity was maintained at 75%±7% with short spikes up to ±15%. Using the interaction between *Arabidopsis* and one of its bacterial pathogens as a test experimental system, we demonstrate that experimental results produced in our chamber were highly comparable to those obtained in a commercial growth chamber. In summary, our design of an inexpensive plant growth chamber will tremendously increase research opportunities in experimental plant biology.

## Introduction

Development, physiology, and responses to stimuli in plants are strongly influenced by environmental conditions. This is known as phenotypic plasticity (e.g., [1–7]). Thus, it is desirable, in experimental plant biology, to contain plant subjects in controlled environments to make reproducible observations. Plant growth chambers are typically used to produce controlled environments. The environmental parameters controlled in growth chambers include temperature, relative humidity (RH), and light cycle. In standard commercial growth chambers, these environmental parameters are precisely controlled, and the control of the parameters can be programmed in a complex manner. But these features come at a price. This financial constraint often determines the maximum affordable size of environment-controlled space and consequently limits the size of some experiments; it makes other experiments infeasible.

We are planning to operate a large-scale undergraduate research program in plant-pathogen interaction studies and need ample environment-controlled space. To overcome the financial constraint associated with commercial growth chambers, we designed and constructed our own growth chamber at a much lower cost. This growth chamber was designed solely for the research purpose described and is not intended to be used commercially or personally.

## Materials and Methods

We built two plant growth chambers with essentially the same design. The biological data produced in and physical data generated by the growth chamber were collected using the first growth chamber. Most photographs of parts and construction procedures of the growth chamber were taken during construction of the second chamber.

### Materials required

All the materials required to construct a growth chamber are listed in S1 Table.

### Requirements for a location

The followings are required for a location to place a growth chamber: (1) a floor space of a 2.5 m (W) x 3.5 m (D) at minimum—a larger space will make the chamber construction easier; (2) a relatively stable ambient temperature within 7°C from the target temperature—note that a heat output of the air conditioner is substantial; (3) an electrical power for 120 VAC 15 A, ideally on a separate circuit; (4) a deionized water line for the humidifier; (5) A drain—a floor drain is ideal; (6) an internet connection for the controller.

### Setting up and programming of the controller

The setup of the Micasaverde Vera 3 controller was performed according to the manufacturer’s instructions. The UI5 user interface was used instead of UI6 for compatibility with some of the apps. We found that including z-wave devices into the z-wave network of the controller was often difficult. This difficulty was overcome by temporarily downgrading the z-wave firmware. The time for the controller is set according to the chosen location. Since we did not want the controller to change the time with Daylight Savings, we chose the St. Thomas Time, which is close to the US Central Time and does not observe Daylight Savings. The wakeup interval and the maximum polling interval (see option “Poll this node at most once every”) for the temperature/RH sensors were set to 60 and 20 seconds, respectively, while the maximum polling interval for the z-wave switches were set 60 seconds. The apps, Program Logic Event Generator (PLEG), Program Logic Core (PLC), Virtual ON/OFF Switches, and dataMine graphing and logging, were installed using the app installation function of UI5. The license for PLEG and PLC were purchased from the provider according to its instructions. The PLEG programs used for the first and second growth chambers are provided as S1 Text. All the sensor records were logged through the dataMine graphing and logging to an USB flash memory attached to the Vera 3 unit, downloaded to a Windows PC connected to the controller network using WinSCP (http://winscp.net/eng/index.php), and analyzed and visualized by a custom R script.

### Measurement of the light spectrum and intensity

An Apogee Blue-Wave VIS-50 Spectrometer was used to measure the light spectrum and intensity on a growth tier of plant growth chamber #1. The intensity was measured approximately 6 cm above the tier floor due to the size of the detector module. The PAR intensity was measured at 49 locations in a 7 x 7 grid on the tier. A 6^th^ order polynomial regression in the two position coordinates was fit to the measured intensity to smooth the spatial PAR intensity distribution and to extrapolate the distribution to the edges of the tier. The data processing and visualization were performed using a custom R script.

### *In planta* bacterial growth assay

The *in planta* bacterial growth assay was performed as described previously [8]. The plants were inoculated with the indicated bacterial strains between 3 and 5 hours after dawn.

## Results and Discussion

### Assumptions factored into the growth chamber design

To reduce the cost, we gave up some of the capabilities commercial growth chambers offer that are not necessary for our experimental needs. First, we assumed that the chamber would have a relatively stable ambient temperature of approximately 22°C and that the temperature required inside the chamber would be within 8°C from the ambient external temperature. These assumptions eliminated requirements of a high level of air separation between inside and outside the chamber, of substantial insulation, and of large heating and cooling capacities.

Second, in many experiments, the levels of parameter control precision offered by commercial growth chambers are not necessary. We assumed that maintaining ±1°C of the target temperature would be sufficient for the temperature control. In an early stage of the growth chamber development, we learned that it would be very difficult to tightly control the RH on a time scale of a few minutes when the air inside the chamber was directly cooled by the evaporator coil of an air conditioner, which functions as a powerful dehumidifier. We also learned that a high level of RH fluctuation in the ambient air (the ambient RH in our experimental setup ranges from 3% in winter to 100% in summer) strongly affects the inside RH as the inside air is not strictly separated from the ambient air. Therefore, to be practical, we assumed that a one-hour average RH within ±8% of the target RH and short-term spikes of the RH (each spike shorter than 5 minutes) within ±15% of the target RH were tolerable. Short-term spikes of the RH lower than the target RH occurred when the cooling cycle of the air conditioner turned on. Short-term spikes of the RH higher than the target RH occurred when the ambient RH was extremely high and when the cooling cycle was off for several minutes. These relaxed requirements in the RH control allowed a simple design of direct air cooling by a portable air conditioner.

Third, we did not require the capability to quickly change the temperature or RH. We assumed that the temperature and RH inside the growth chamber were not required to change faster than at the rates of 1°C in 15 minutes or 10% in 15 minutes, respectively. These assumptions eliminated requirements of larger cooling, heating, humidifying, and dehumidifying capacities and removed the need for a very high air flow rate inside.

To ensure that construction of growth chambers of the same design would be feasible for many people, we only used materials that can easily be purchased online or from local hardware stores in the US. The total material cost was $2,300 for one growth chamber. The comprehensive parts list, including venders we used, is included as S1 Table.

### The structure

**Fig 1:**
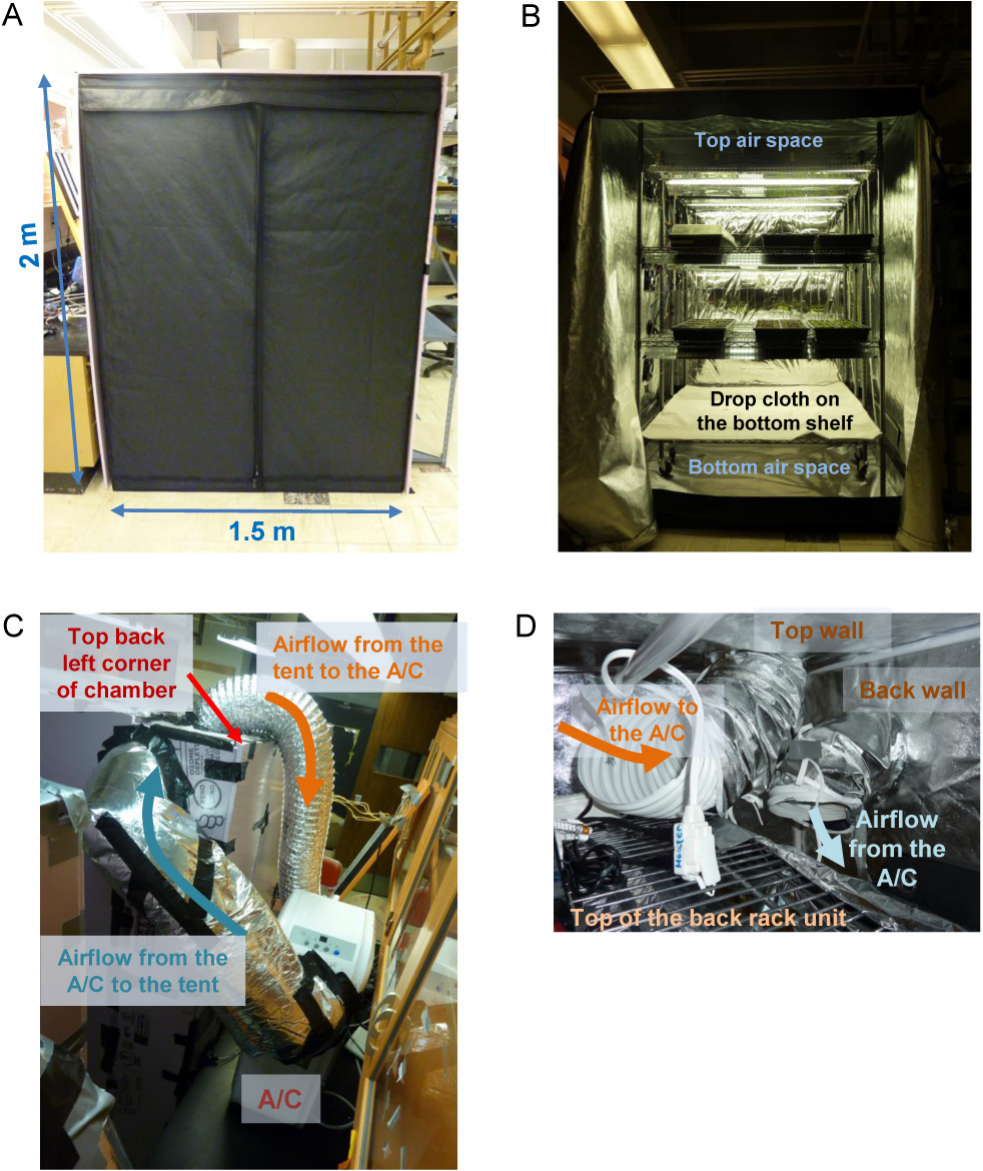
External and interior views of the assembled growth chamber. A. Front view. The outside dimensions of the growth tent were 1.5 m (W) x 1.5 m (D, not shown) x 2 m (H). The sides, the top, and the back (not shown) of the growth tent were covered with pink insulation boards. B. Front view with the tent open. A drop cloth was spread on the bottom shelf to control air flow. Planting flats are shown on the top and middle growth tiers. The top and bottom air spaces are indicated. C. Outside view from behind the chamber looking towards the top back left corner of the growth chamber. The air conditioner and the air ducts that connect the air conditioner and the tent are shown. Note that the air duct for returning air from the air conditioner to the tent is insulated. A/C, air conditioner. The left and right sides of the growth chamber are defined by the view from the front. D. Inside view from the top air space looking towards the top back left corner of the growth chamber. The ends of the air ducts that connect to the air conditioner are shown. Note that the air going to the air conditioner is collected from the top air space and that the air returning from the air conditioner is directed diagonally to the bottom back right corner across the air mixing space in the back side of the chamber. Part of the top shelf of the rear rack unit and the extension cord for the night-time compensation heaters are also shown.

**Fig 2:**
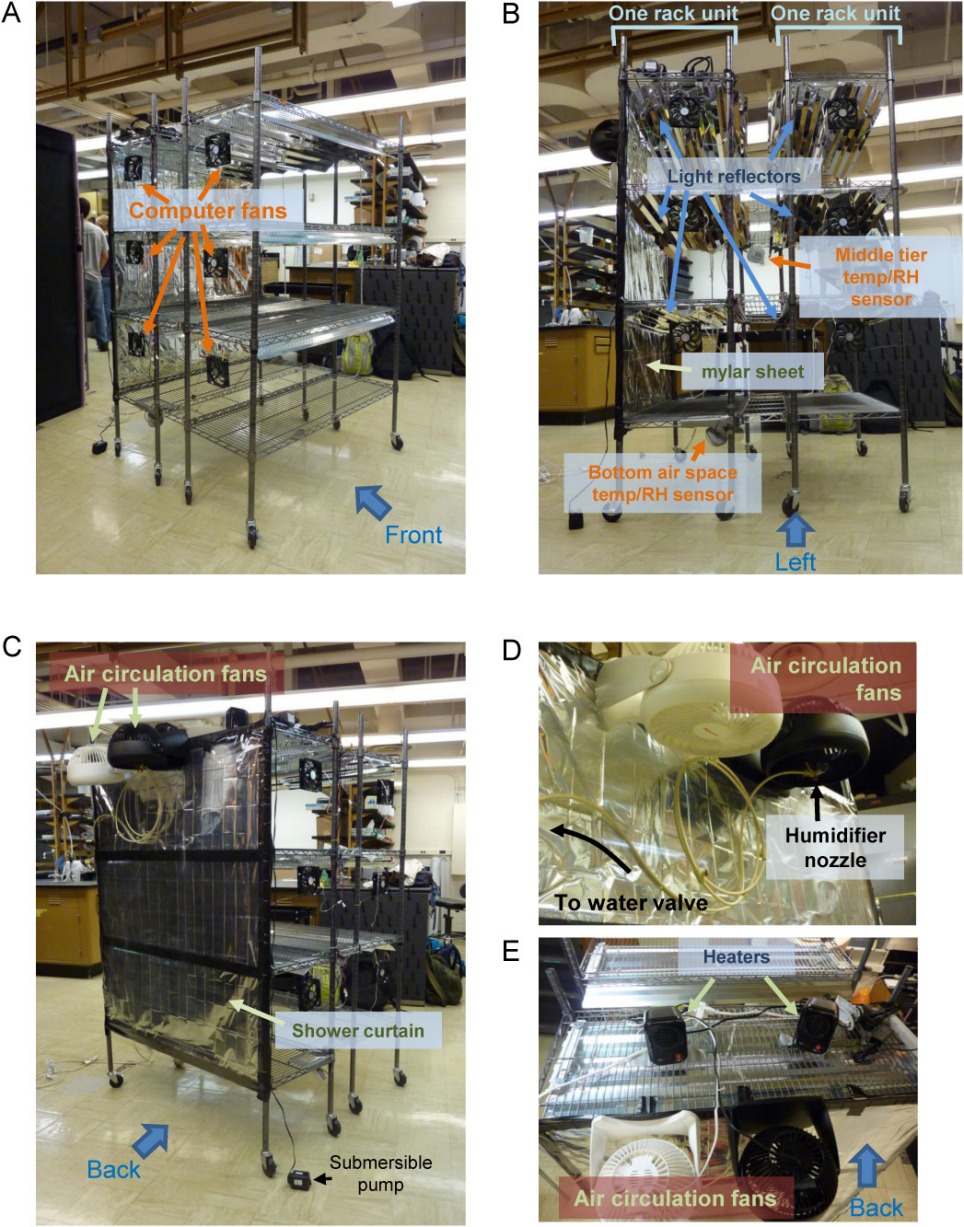
The wired racks with parts attached to the racks. A. View from the front left corner; B. View from the left side; C. View from the back left corner. The wired racks and the attached parts were fully assembled outside the growth tent and then rolled into the tent, with the exception of four parts: three narrow shelves connecting the two rack units and the middle growth tier sensor that was hung from the top narrow shelf were inserted once the racks were in the tent. Once the racks were in the tent, the drainage system then assembled in the tent. Note that the submersible pump that was placed inside the plastic tub in the bottom air space is shown loose on the floor in these photographs. Then the narrow shelves and the middle growth tier sensors were attached, and electrical wiring, including wiring for the lights, was finished. D. Close up view of the humidifier nozzle and air circulation fans. The fans were attached to the top of the air mixing space in the back of the chamber; this space is separated from the growth space by a Mylar sheet and a shower curtain liner as shown in B and C. E. Top view of the racks, taken from the back. Two desktop heaters were placed on top of the racks to provide compensating heat when the lights were off (these are the night-time compensation heaters). Power adaptors for the computer fans (shown in A), the temperature/RH sensors (shown in B), and the submersible pump (shown in C) were also placed on the top shelf of the rear rack unit; these components are partly seen on the right side of the figure.

The outside shell of the growth chamber was made of a plant growth tent (outside diameter, 1.5 m (W) x 1.5 m (D) x 2 m (H)), which was practically light tight and had reflective inside walls (Fig 1A and 1B). Wired racks were placed inside the plant growth tent to provide growth tiers. Since we used the growth chamber to grow Arabidopsis, and since Arabidopsis does not require a tier height of more than 40 cm, we made three growth tiers (Figs 1B and 2), each of which had an area dimension of 1.2 m by 1.2 m. We typically use 60 cm x 30 cm plastic flats to grow Arabidopsis. Each tier can hold eight such flats, which makes each tier two flats deep. It could be inconvenient because flats in the front row need to come out to reach flats in the back row. However, we prioritized in the growth chamber design for size of the growth space for the budget over this potential inconvenience. For growth of other plant species, the wired rack growth tiers can be arranged flexibly as needed.

The left, right, back, and top sides of the growth tent were covered with ½-inch thick insulation foam boards to moderately insulate the inside of the tent from the ambient temperature (Fig 1A and 1C).

### The air flow design

**Fig 3:**
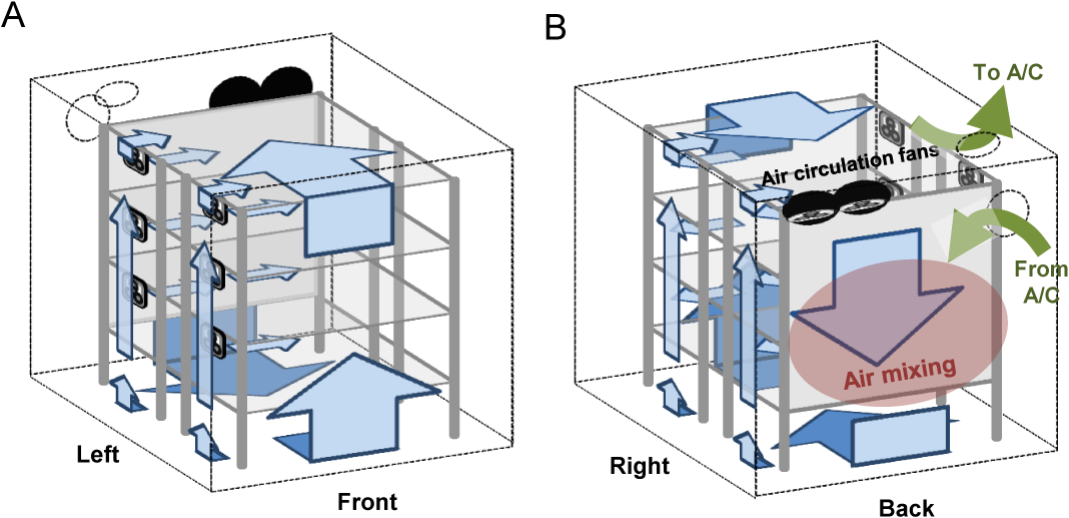
Schematics of the air flow design. A. Front view. B. Rear view. Wired racks and attached fans are drawn; dotted lines outline the growth tent. Inside air flows are shown by blue arrows. The bulk of the air flow occurs along the periphery of the tent interior. Bulk air flow is mainly driven by two air circulation fans placed at the top of the rear air mixing space. In B, air flows from and to the air conditioner (A/C) are highlighted, as well as the space where air from the A/C and the inside air are mixed.

Air coming out of our air conditioner was very cold and dry. Such cold air with very low RH should not be blown on plants. We relatively isolated the back part of the inside of the growth tent (approximately 30 cm deep) by putting a shower curtain on the back side of the wired racks to use the back part for air mixing (Fig 2C). A reflective Mylar sheet was attached on the plant growth space side of the shower curtain to help with homogenizing the light intensity on the growth tiers (Fig 2B). Initially, we only used a Mylar sheet for the purpose of isolating the air mixing space, but the Mylar sheet was degraded by constant exposure to water from the humidifier and was eventually torn in a few months. Thus, we added a shower curtain on the air mixing space side. In the air mixing space, two air-circulation fans blew the inside air downward from the top (Figs 2C, 2D, and 3). The air coming out of the air conditioner was directed diagonally from the top left corner to the bottom right corner of the air mixing space (Figs 1D and 3B), which allowed quick mixing of the air from the air conditioner with the inside air. The mixed air was directed to the space below the bottom growth tier (the bottom air space) for further mixing. We left 35 cm below the bottom growth tier open for this purpose. A drop cloth was spread on the shelf of the bottom growth tier, so that the bottom air space was relatively isolated from the bottom growth tier. The air from the bottom air space was directed up along the front and side walls of the growth tent to the top air space which was 24 cm high, and the air was circulated again through the fans at the top of the air mixing space (Fig 3). Part of the air in the top air space was also fed to the air conditioner to be cooled (Fig 1D). This peripheral air flow inside the growth tent was designed to be at a relatively high rate to keep the peripheral air fairly homogenous in temperature and RH. A relatively small portion of the peripheral air flow along the side wall was brought into each growth tier space by computer cooling fans to supply the growing plants air with controlled temperature and RH and to remove the heat generated by the lights in each tier.

### The lights

**Fig 4:**
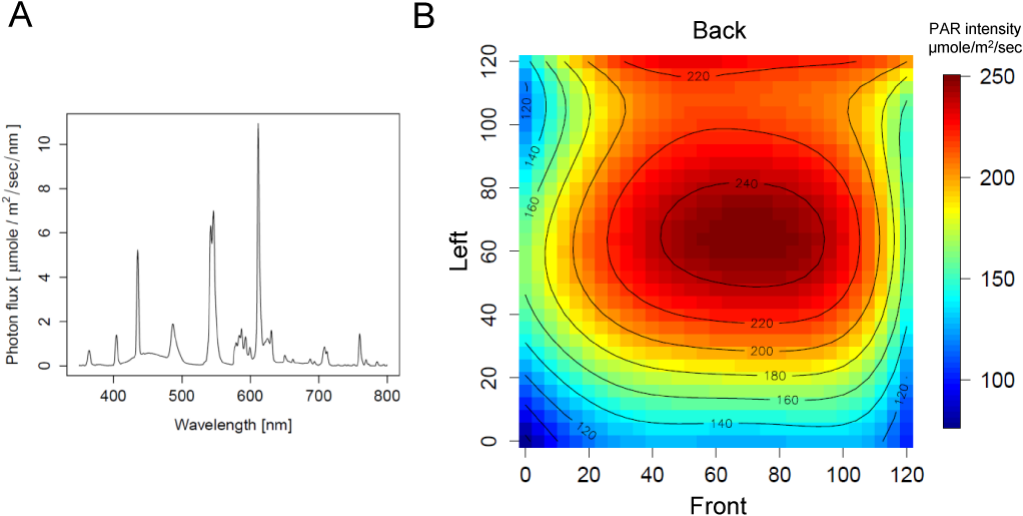
Characterizing chamber lighting. A. Light spectrum of the F54T5/841 HO fluorescent bulb. The spectrum was recorded at approximately the center of the bottom tier, and the intensity of the photosynthetically active radiation (PAR, 400 to 700 nm) was 243.8 μmole/m^2^/s. B. Spatial distribution of the PAR intensity on the bottom growth tier (a 120 cm x 120 cm area). 2-D polynomial regression model-predicted PAR values were used to generate the colored contour plot. ‘Front’, ‘Back’, and ‘Left’ of the tier are indicated. The values shown along the bottom and the left edges of the plot indicate the positions on the tier in cm. The color code for the PAR intensity is shown on the right of the plot. When these measurements were made, the air temperature of the bottom tier was 22°C, and the bottom and middle tiers had no flats. When the middle tier was loaded with flats to block stray light from the top and middle tiers coming through the gap between the light reflectors for the bottom tier (see Fig 2B), the PAR intensity was decreased by 6% at *x* = 60, *y* = 60, 2% at *x* = 60, *y* = 25, and 1% at *x* = 60, *y* = 95 (*x* and *y* coordinates are according to those in B).

**Fig 5:**
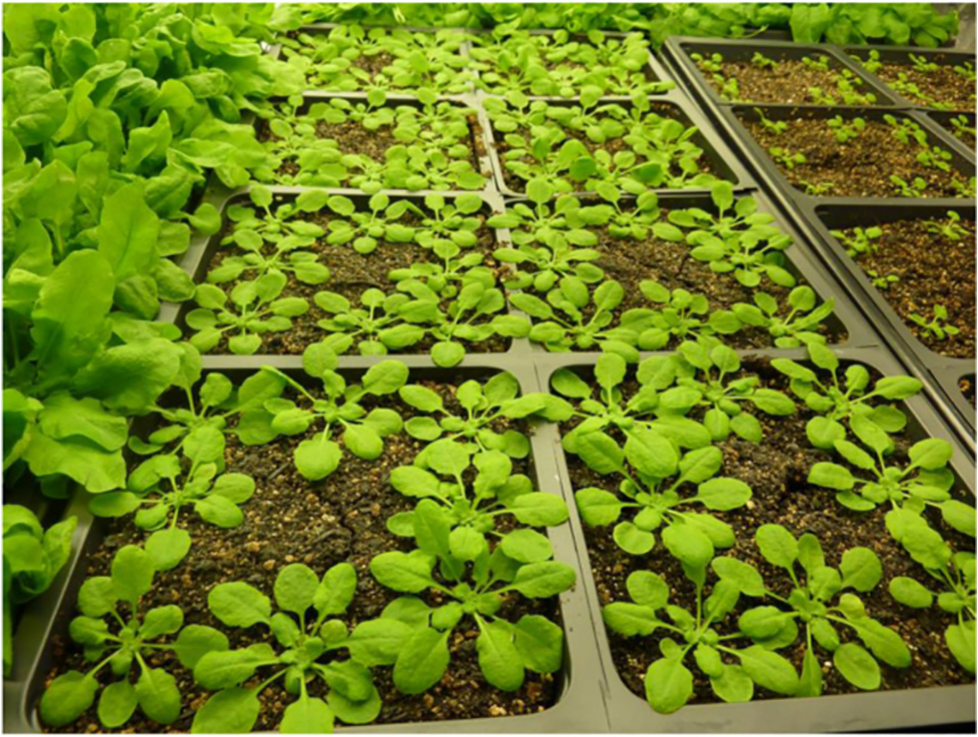
Arabidopsis plants grew well in the growth chamber. Shown in the middle are five-week old *A*. *thaliana* plants, accession Col-0, wild type. While plants were growing, chamber controls were set at 22°C and 75% RH; lights were on a 10.5-hour light/13.5-hour dark cycle.

We used four 54 watt, 1.2 meter T5 high-output fluorescent lamps (F54T5/841 HO) per tier, together with large reflectors (Fig 2B), to supply relatively homogenous lighting. Fig 4A shows the spectrum of the light produced. Fig 4B shows the spatial distribution of the photosynthetically active radiation (PAR, 400 to 700 nm) intensity on one growth tier. The PAR intensity in most of the tier ranged from 140 to 250 μmoles/m^2^/sec. The spectrum and the intensity of the lights were appropriate for Arabidopsis growth (Fig 5). The reason that the PAR intensity was lower near the front end compared to that near the back end (Fig 4B) is likely because the back side of the tier had a highly reflective Mylar sheet, which was placed at the back edge of the tier. Consistent with this, the PAR intensity was higher at the back edge than a little away from the back edge. The front end had the inside material of the growth tent, whose reflection coefficient is lower than that of a Mylar sheet, which was located approximately 10 cm away from the front edge of the tier. Since we needed access to the growth tiers, we could not place a highly reflective material at the front edges of the tiers. Modification of the light reflector shape may moderate the heterogeneous spatial distribution of the PAR intensity. Since the PAR intensity is sufficiently high, use of light diffusers may also be an option.

To reduce the total heat generation inside the growth tent, electronic ballasts for the fluorescent lamps were placed outside the tent. Some plants may need particular wavelengths of light, such as infra-red, supplemented for their growth. In such a case, the amount of heat generated by the supplemental light sources need to be considered.

### The night-time compensation heaters

Since our target temperature was close to the ambient temperature, the temperature inside was not very stable without any substantial heat generation inside, i.e., when the lights were off. For this reason, we placed two 200 watt desktop heaters in the top air space to compensate the heat when the lights were off (Fig 2E).

### The cooling system

Cooling was provided by a portable air conditioner (Soleus Air KY-80), which had outside dimensions of 30 cm (W) x 35 cm (D) x 79 cm (H). It had a maximum cooling capacity of 2,300 watts (8,000 BTU), which provided ample cooling capacity compared with the total heat amount generated inside the growth tent. This extra capacity allowed the inside temperature to be set more than 9°C lower than the ambient temperature even when the lights are on. The air conditioner was placed outside the growth tent, which made the footprint of the chamber a little larger than the footprint of the growth tent. The air from the growth tent was drawn into the air intake for the upper chamber of the air conditioner (which had the evaporator coil), and the cooled air coming out of the upper chamber was sent back to the growth tent (Fig 1C). The duct that sent the air back from the air conditioner to the growth tent was insulated with a fiber-glass sleeve to avoid ambient heating of the cooled air and to avoid condensation outside the duct when the ambient air is humid.

To control the air conditioner by a z-wave switch (see below for z-wave), the negative temperature coefficient thermistor used for the air temperature sensor of the air conditioner was replaced with a relay that could be electronically switched between 30 kΩ and 6 kΩ resistors. The 30 kΩ and 6 kΩ resistors mimicked the sensor state for low and high temperatures, respectively. Switching to the 6 kΩ resistor turned on the cooling cycle of the air conditioner when the air conditioner was set for the “auto” mode. This allowed us to directly control when the air-conditioner entered and exited a cooling cycle, rather than being forced to rely on the air conditioner’s inbuilt controls. The auto mode also kept the fan of the air conditioner running all the time whether the cooling cycle was on or off.

### The humidifier

Since the air conditioner was a powerful dehumidifier, a powerful humidifier was required to counter it. Ordinary commercial room humidifiers were not sufficiently powerful for this purpose. We used a cooling water mister that was designed to decrease temperature outside in summer by evaporation of water mist. Three of the four nozzles of the mister were clogged with silicon caulk, and the remaining one nozzle was used. The humidifier made of just one nozzle was powerful enough to increase the RH even when the cooling cycle was on. The mister nozzle was placed in the middle of one of the two air circulating fans in the air-mixing space (Fig 2D). The humidifier was controlled by an electrical water valve placed in the water line to the nozzle, and the electrical water valve was controlled by a z-wave switch. Deionized water was used for the humidifier to avoid sediment accretion when water evaporated.

Not all water mist generated by the mister nozzle evaporated in the small air-mixing space even with the high air flow. Water that was not evaporated accumulated right under the air circulation fan blowing the mist. If the space in which the growth chamber was placed had been equipped with a floor drain, this would not have been a problem. However, without a floor drain we had to collect and remove the water. We placed a plastic tub (a plastic storage box without a lid) under the fan to collect water, and the water accumulated in the tub was drained by a submersible fountain pump with auto shut off. Condensation water from the air conditioner also needed to be drained, so we drained it into the plastic tub as well.

### The control system

The entire growth chamber system was controlled by a z-wave technology-based controller, Micasaverde Vera 3. The controller was wirelessly connected via z-wave to two temperature/humidity sensors and four switches. The controller received temperature and humidity readings from the sensors approximately every minute and controlled the switches’ on/off states. With this temperature/RH sampling frequency, the AA batteries used in the sensor were used up rather rapidly (~1 month). Therefore, we wired the sensors to a 120 V AC voltage supply through a 4.5 V DC power adaptor. One z-wave switch was used to switch the relay that replaced the air temperature sensor of the air conditioner; this switch turned the cooling cycle on or off. Another switch was used to switch on or off the electrical water valve for the humidifier. The third switch was used to switch the lights on or off according to the controller’s clock, and the fourth switch was used to switch the night-time compensation heaters on or off also according to the controller’s clock.

The controller can be programmed and monitored by a computer under the controller’s network (the controller is also a WiFi network router), or by accessing, via internet, the supplier’s server which frequently communicates with the controller. There is no extra fee to use the MiCasaVerde internet tools to manage a Vera 3.

A drawback of the Vera 3 controller was that it only uses integer values for temperature and RH although the sensors can determine values to one digit below decimal point. To control temperature more precisely we used the Fahrenheit temperature mode of the controller instead of the Celsius mode because 1 degree in Fahrenheit is 0.56 degrees in Celsius.

### Control of the environmental parameters and the program for the controller

We had multiple challenges in controlling the inside temperature and RH. First, since only a small portion of the peripheral circulating air was brought into each growth tier, the temperature and RH readings from a growth tier were delayed compared to temperature and RH changes in the peripheral circulating air. Thus, the temperature and RH control using the temperature and RH readings from a growth tier would overshoot the temperature and RH at the growth tier. We substantially moderated this problem by placing a second temperature/RH sensor in the bottom air space (Fig 2B). The readings from this second sensor were used to switch the cooling cycle and the humidifier.

Second, the lights were the major heat source when they were on, and they were located in each tier. The night-time compensation heaters, the major heat source when the lights were off, were located in the top air space. Thus, difference in the temperature between a tier and the peripheral circulating air changed according to whether the lights were on or off. We empirically determined this temperature difference when the lights were on or off and offset the target temperature at the second sensor in the bottom air space accordingly. Our target temperature at a growth tier was 22.2°C (72°F), thus we set the bottom space sensor to 20°C when the lights were on and 21.7°C when the lights were off. Since the RH increases approximately 5% when the moisture amount in the air is constant and the temperature decreases by 1°C around these temperatures, the target RH at the bottom air space sensor needed to be offset as well. For our target RH of 75% at 22°C in a growth tier, the target RH at the bottom air space sensor was set for 83% at 20°C when the lights were on and 77% at 21.5°C when the lights were off.

A third challenge: the controller did not collect temperature and humidity readings more frequently than approximately every minute. The air conditioner was a powerful dehumidifier, and it could decrease the RH by approximately 10% in a minute when the humidifier was off. Since the humidifier was powerful enough to counter the air conditioner’s dehumidification power, the humidifier could increase the RH approximately 10% in a minute when the cooling cycle was off. With this low data collection frequency from the sensor, the RH would easily overshoot on both the higher or lower ends of the desired range. We implemented a feature in the program to substantially moderate this problem. We programmed the humidifier switching with different threshold RHs according to whether the cooling cycle was on or off and according to whether the lights were on or off. (The light status changed the threshold temperature, and consequently the threshold RH.)

Fourth, the behavior of the inside RH change was strongly affected by variable ambient RH. For example, when the cooling cycle was off, the inside RH increased in summer, as the ambient RH was higher than the target RH (ambient RH > 90%) and decreased in winter, when the ambient RH was lower than target RH (ambient RH < 10%). The behavior of the inside RH change was also affected by the quantity of plants in the growth chamber. To compensate for the variability of the ambient RH and the effect of the plants inside, four different sets of threshold RHs were made: Set 1 for the highest ambient RH to Set 4 for the lowest ambient RH. Then a threshold RH set was selected according to the RH reading by the middle growth tier sensor right after the cooling cycle was turned on. This RH reading likely represents the impact of the ambient RH and the inside plant quantity well because it was collected after a period when the cooling cycle had been off; the RH reading had recovered from dehumidification by the previous cooling cycle. Table 1 shows the threshold RH values according to cooling and light states in the four different RH threshold sets (Sets 1–4). Immediately after the cooling cycle turned on, the RH threshold set was re-selected according to the tier RH value.

**Table 1:** Program sets to adjust the RH threshold according to the effects of the ambient RH and the inside plant quantity.

Program name	Set 1	Set 2	Set 3	Set 4
Virtual switch linked	Hum1Vs1	Hum1Vs2	Hum1Vs3	Hum1Vs4
RH range at middle tier sensor after cooling cycle, above	85	77	71	-
RH range at middle tier sensor after cooling cycle, below	-	86	78	72
RH value at bootom air space to have humidifier on, lights on, cooling on, below	84	86	88	90
RH value at bootom air space to have humidifier on, lights on, cooling off, below	68	72	76	78
RH value at bootom air space to have humidifier on, lights off, cooling on, below	76	78	80	82
RH value at bootom air space to have humidifier on, lights off, cooling off, below	61	65	68	72

We used the following apps for Vera 3: PLEG (http://rts-services.com/Vera/Plugin/PLEG/), PLC (http://rts-services.com/Vera/Plugin/PLC/), which was required for PLEG, and Virtual ON/OFF Switches (https://apps.mios.com/plugin.php?id=1408). Four virtual switches, which were used for our four RH threshold sets, were made by Virtual ON/OFF Switches. The entire set of programs used in PLEG is included in S1 Text. We also used another app, dataMine graphing and logging (http://code.mios.com/trac/mios_datamine), to log the sensor readings and the state of the switches in the Vera 3 unit.

Although we did not implement this feature in the program, it is possible to program emergency shut down and warning. For example, if the chamber should shut down upon reaching a threshold high temperature, the heat sources, the lights and the night-time compensation heater can be programmed to be turned off when the middle growth tier temperature is over a threshold value. It is also possible to set the controller to send an e-mail message upon detection of this condition.

One thing we should emphasize is that no two growth chambers will be exactly the same when they are built with consumer products that are not specifically designed and produced for the purpose of building our growth chambers. We needed to use somewhat different temperature and RH threshold values in the PLEG programs for the two growth chambers we constructed (S1 Text). Growth chamber control relies on hand-tuning an intricate computer control program to compensate for hardware weakness introduced by our cost-cutting measures.

### Skills and labor required to build a growth chamber

Since the growth chamber was made from consumer products, for the most part no special skills were required to put together a growth chamber. However, basic do-it-yourself skills, particularly basic electrical skills, would definitely help to construct a growth chamber efficiently. We estimate that two adults with basic do-it-yourself skills would not need more than three days to put together a growth chamber if they have carefully read through the procedures here and if all the materials are in hand before starting construction. Once the growth chamber is assembled, it may take some time to empirically optimize temperature and RH threshold values in a controller program, as no two growth chambers built will be exactly the same. If the desired target temperature and RH are different from those we used, the parameter values in the program need to be optimized through trial and error: change corresponding parameter values, run the chamber for a few days, monitor how the chamber has operated (The app DataMine graphing and logging is convenient for this purpose), and repeat this cycle until the chamber operation is satisfactory. This program optimization may take some time (up to a few weeks) although very little labor is required during the period of optimization.

For consistent operation, yearly maintenance of the growth chamber is recommended. The yearly maintenance includes: replace the humidifier mist nozzle with a new one (we found that the plastic connector of the mister model we used could crack after a while, so replacing the entire mister yearly may be a good idea); inspect parts that get wet from humidifier mists and replace them if needed (for example, steel wires used to fix the nozzle to a fan may get rusty); clean the plastic tub that collects excess water from the humidifier and the submersible pump in the tub as they accumulate “scum”; check taped parts and redo them if needed (particularly, tapes used for fixing air ducts and tapes used to seal the back bottom part of the tent); replace fluorescent lamps if needed.

### Actual temperature and RH control

**Fig 6:**
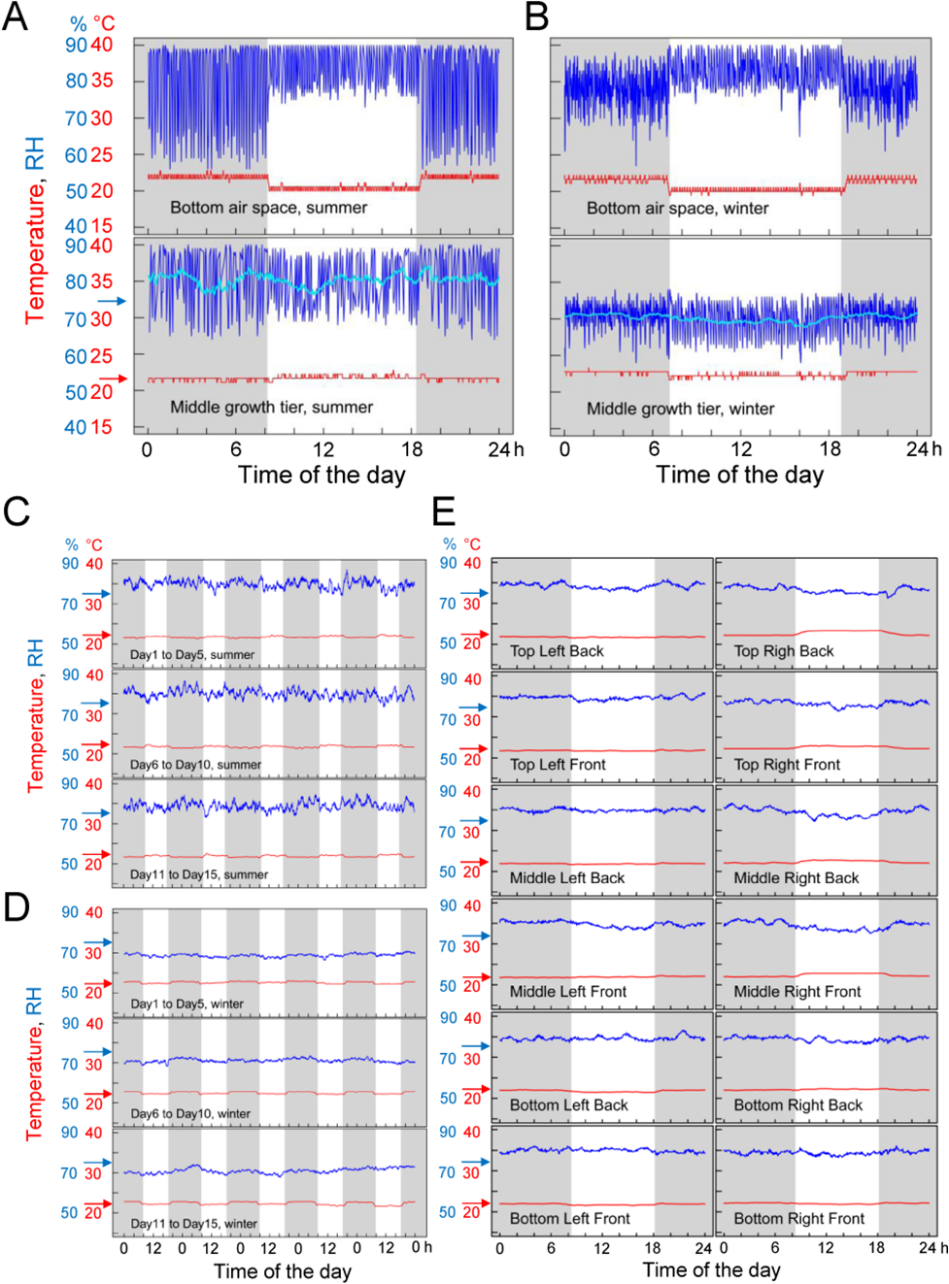
The temperature and RH inside the growth chamber were well controlled. A and B. Records from one summer day (A) and one winter day (B) of the temperature (red) and RH (blue) by the sensor in the bottom air space (top) and the sensor in the middle growth tier(bottom). In the middle growth tier, the 1 hour average RH calculated from a 1 hour sliding window is shown in light blue. The sensors saturate at 90% RH (evident in the bottom air space RH measurements during the day in A). However, middle growth tier RH measurements rarely reached 90%, so the 1 hour average RH values are accurate. C. and D. Fifteen consecutive summer-day (C) and winter-day (D) records of the 1 hour averaged temperature (red) and RH (blue) measured by the middle growth tier sensor. E. One-day records of the 1 hour averaged temperature (red) and RH (blue) at different spots in the growth space in summer. On each growth tier (“Top”, “Middle”, and “Bottom”), a sensor was placed in the middle of each of four quadrants (“Front Right”, “Back Right”, “Back Left”, and “Front Left”). Note that a single sensor was moved from spot to spot to record the temperature and RH, so the measurements for different spots were collected on different days. Gray background shading in the plots indicates times when the lights were off, and a white background indicates when the lights were on. Light/dark cycles for summer and winter were different because different experiments performed in the chamber during these times required different lighting regimens. Arrows on the left of each plot shows the target temperature, 22°C (red), and target RH, 75% (blue). When these measurements were made, there were two to six flats of plants in each tier. The soil temperature (~3 cm deep from the top surface of the soil) in a flat was not significantly different from the air temperature of the growth tier.

Fig 6A and 6B show a one-day record of temperature and RH readings from the sensors placed in the bottom air space and in the middle growth tier, recorded in summer (mid July, 6A) and in winter (mid January, 6B). These dates were chosen to demonstrate the growth chamber performance when the ambient RH was the highest (summer) and the lowest (winter). The temperature in the middle growth tier was always within 1°C of the target temperature of 22°C in both seasons, which satisfied our design goal. The temperature in the bottom air space experienced an offset according to the state of the lights; this offset had been deliberately introduced to make the overall temperature in the growth tiers as constant as possible. The RH in both the bottom air space and the middle growth tier showed short spikes of the RH caused by the cooling cycle, the humidifier, and the ambient RH. The spikes of the RH in the middle growth tier were generally between +15% and -10% in summer and between +2% and -13% in winter of the target RH of 75%. The 1 hour average RH slowly varied between +1% and +8% in summer and between -4% and -6% in winter of the target RH of 75%. Adjusting the RH threshold values, including the RH value ranges that determine when the program will switch RH threshold sets, should improve these 1 hour average RH deviations from the target RH. We did not attempt to search for the best RH threshold values since we had plant biology experiments underway, and we did not want to change the environmental parameters part way through these experiments. Even though we did not fully optimize the current program, the growth chamber RH control satisfied our design goal.

The 1 hour average temperature and RH data in the middle growth tier across 15-day spans are shown in Fig 6C (summer) and 6D (winter). These measurements demonstrate consistent performance of the growth chamber throughout bi-monthly periods.

Fig 6E shows the 1 hour average temperature and RH data for one day in summer in different spots of the growth space, demonstrating no obvious difference in the temperature and RH among the three growth tiers.

### Plants interactions with bacterial pathogens

**Fig 7:**
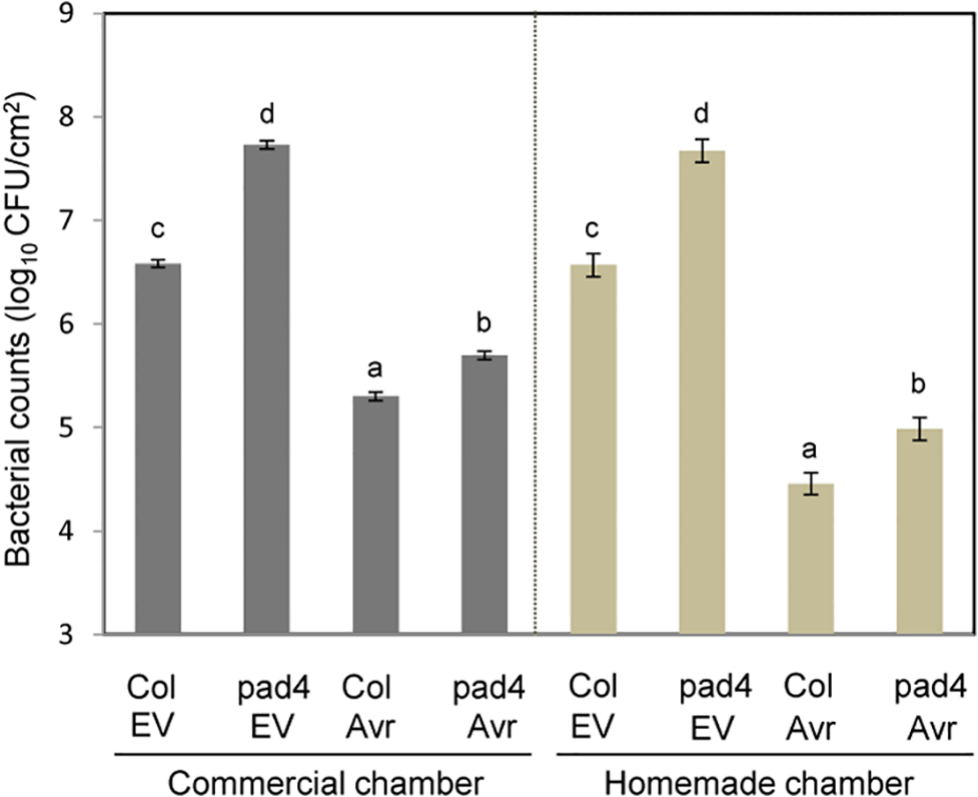
*In planta* growth of bacterial pathogen strains was consistent between the homemade growth chamber and a commercial growth chamber. Wild type (Col) and *pad4-1* mutant plants of the *Arabidopsis thaliana* accession Col-0 were inoculated with the *Pseudomonas syringae* pv. *tomato* DC3000 (*Pto*) strain carrying a vector with the effector gene AvrRpt2 (Avr) or the *Pto* strain carrying the empty vector (EV).The bacterial inoculation dose was 0.0002 OD_600_. The bacterial colony forming units (CFU) were quantified 3 days after inoculation. Four-week old plants were grown at 22°C with a RH of 75% and a 12 hour light/12 hour dark cycle. The experiment was repeated three times in both the commercial and the homemade growth chambers. In each experiment at least 8 biological replicates were made. Bacterial growth results for the two growth chambers were analyzed separately using mixed-effects linear model. The error bars indicate standard errors for the mean values. Bars with different letters are significantly different at *p* < 0.01.

Interactions between plants and pathogens are often highly affected by environmental conditions (e.g., [1–3,6]). We compared interactions between Arabidopsis and its bacterial pathogen *Pseudomonas syringae* in a commercial chamber (Conviron BDR16) and one of our homemade chambers. Two genotypes of plants, the wild-type Arabidopsis accession Col-0 and a mutant with Col-0 background, *pad4-1*, were used. Plants were grown at growth chamber settings of 22°C and RH of 75% with a 12-hr light/12-hr dark cycle. Leaves of four-week old plants were inoculated with *P*. *syringae* pv. *tomato* DC3000 carrying either an empty vector (EV) or the same vector carrying the effector gene *AvrRpt2* (Avr) at a dose of OD_600_ = 0.0002, and the bacterial titer was quantified 3 days after inoculation (Fig 7). The bacterial growth in these four plant genotype and bacterial strain combinations were very similar between the commercial and homemade growth chambers: the rank-ordering of bacterial growth among the four plant genotype and bacterial strain combinations, Col and Avr, pad4 and Avr, Col and EV, and pad4 and EV, was consistent between two growth chambers, and the differences among the four plant genotype and bacterial strain combinations were all significant (*p* < 0.01). Thus, interactions between Arabidopsis and *P*. *syrinage* were highly comparable between a commercial growth chamber and our growth chamber.

## Conclusions

We demonstrated that it is feasible to build an inexpensive plant growth chamber from consumer products, which can provide a well-controlled environment, defined by the temperature, RH, and the light intensity and cycle. Arabidopsis plants, before bacterial inoculation, grow with no apparent stress symptoms in the growth chamber. Experimental results for the interactions between Arabidopsis and *P*. *syringae* were highly comparable between a commercial growth chamber and one of our homemade growth chambers. We provide detailed information about how to build a plant growth chamber of the same design. Our homemade growth chamber design will vastly enhance research opportunities in experimental plant biology by providing, at very low-cost, the controlled growth environments critical for plant biology research.

## Supporting Information

S1 Text The PLEG program for controlling the plant growth chamber. (DOCX)

S1 Table List of materials used for construction of a plant growth chamber. (XLSX)
