# Supplementary material for: Design and Construction of an Inexpensive Homemade Plant Growth Chamber
Source: PLoS One. 2015 May 12;10(5):e0126826. doi: 10.1371/journal.pone.0126826 (PMC4428828; doi:10.1371/journal.pone.0126826)
Supplement: S1 Text — (DOCX) [file pone.0126826.s001.docx]

**Supplemental Text 1:**

**Program Logic Event Generator (PLEG) program for controlling the plant growth chamber**

The following is the Report from the app PLEG, which shows the program used. Two temperature/RH sensors were used, but they are not distinguished in the Report. Among the Triggers, those starting with “TempDay”, “TempNight”, and “Hum1C” show the trigger settings for the controlling temperature/RH sensor, which located in the bottom air space of the growth chamber (“second sensor”). Those starting with “Temp1M” and “Hum1M” show the trigger settings for the monitoring temperature/RH sensor, which located in the middle growth tier (“first sensor”). “Hum1Test” and “Hum1Vs1” to “4” are virtual switches generated using the app Virtual ON/OFF Switches. The other devices used were four z-wave-controlled switches: “Cooling1”, which switches the air conditioner cooling cycle; “Humidity 1”, which switches the water valve for the humidifier; “Lights1”, which switches the power to the ballasts for the fluorescent lamps; “NightHeater1”, which switches the power to the night time compensation heaters.

(1) The program for the first growth chamber.

**Triggers**

| **Name** | **Description** |
| --- | --- |
| Cooling1IsON | Cooling1 is turned on |
| Hum1CA60 | Humidity reported by _Embedded Humidity Sensor go above 60 |
| Hum1CA64 | Humidity reported by _Embedded Humidity Sensor go above 64 |
| Hum1CA67 | Humidity reported by _Embedded Humidity Sensor go above 67 |
| Hum1CA71 | Humidity reported by _Embedded Humidity Sensor go above 71 |
| Hum1CA75 | Humidity reported by _Embedded Humidity Sensor go above 75 |
| Hum1CA77 | Humidity reported by _Embedded Humidity Sensor go above 77 |
| Hum1CA79 | Humidity reported by _Embedded Humidity Sensor go above 79 |
| Hum1CA81 | Humidity reported by _Embedded Humidity Sensor go above 81 |
| Hum1CA83 | Humidity reported by _Embedded Humidity Sensor go above 83 |
| Hum1CA85 | Humidity reported by _Embedded Humidity Sensor go above 85 |
| Hum1CA87 | Humidity reported by _Embedded Humidity Sensor go above 87 |
| Hum1CA89 | Humidity reported by _Embedded Humidity Sensor go above 89 |
| Hum1CB61 | Humidity reported by _Embedded Humidity Sensor go below 61 |
| Hum1CB65 | Humidity reported by _Embedded Humidity Sensor go below 65 |
| Hum1CB68 | Humidity reported by _Embedded Humidity Sensor go below 68 |
| Hum1CB72 | Humidity reported by _Embedded Humidity Sensor go below 72 |
| Hum1CB76 | Humidity reported by _Embedded Humidity Sensor go below 76 |
| Hum1CB78 | Humidity reported by _Embedded Humidity Sensor go below 78 |
| Hum1CB80 | Humidity reported by _Embedded Humidity Sensor go below 80 |
| Hum1CB82 | Humidity reported by _Embedded Humidity Sensor go below 82 |
| Hum1CB84 | Humidity reported by _Embedded Humidity Sensor go below 84 |
| Hum1CB86 | Humidity reported by _Embedded Humidity Sensor go below 86 |
| Hum1CB88 | Humidity reported by _Embedded Humidity Sensor go below 88 |
| Hum1CB90 | Humidity reported by _Embedded Humidity Sensor go below 90 |
| Hum1MA71 | Humidity reported by _Embedded Humidity Sensor go above 71 |
| Hum1MA77 | Humidity reported by _Embedded Humidity Sensor go above 77 |
| Hum1MA85 | Humidity reported by _Embedded Humidity Sensor go above 85 |
| Hum1MB72 | Humidity reported by _Embedded Humidity Sensor go below 72 |
| Hum1MB78 | Humidity reported by _Embedded Humidity Sensor go below 78 |
| Hum1MB86 | Humidity reported by _Embedded Humidity Sensor go below 86 |
| Hum1TestIsON | Hum1Test is turned on |
| Hum1Vs1IsON | Hum1Vs1 is turned on |
| Hum1Vs2IsON | Hum1Vs2 is turned on |
| Hum1Vs3IsON | Hum1Vs3 is turned on |
| Hum1Vs4IsON | Hum1Vs4 is turned on |
| Lights1IsON | Lights1 is turned on |
| Temp1MA73 | _Embedded Temperature Sensor temperature goes above 73 degrees |
| Temp1MB71 | _Embedded Temperature Sensor temperature goes below 71 degrees |
| TempDayA1 | _Embedded Temperature Sensor temperature goes above 68 degrees |
| TempDayB1 | _Embedded Temperature Sensor temperature goes below 69 degrees |
| TempNightA1 | _Embedded Temperature Sensor temperature goes above 71 degrees |
| TempNightB1 | _Embedded Temperature Sensor temperature goes below 72 degrees |

**Schedules**

| **Name** | **On Type** | **On Time** | **On Days** | **Random On Delay** | **Off After Type** | **Off Time** | **Off Days** | **Random Off Delay** |
| --- | --- | --- | --- | --- | --- | --- | --- | --- |
| Sunrise1 | Weekly | 09:00:00 | 1,2,3,4,5,6,7 | 30 | Weekly | 19:30:00 | 1,2,3,4,5,6,7 | 30 |
| Sunrise1sub | Weekly | 09:10:00 | 1,2,3,4,5,6,7 | 30 | Weekly | 19:40:00 | 1,2,3,4,5,6,7 | 30 |

**Conditions**

| **Name** | **Repeat** | **Expression** |
| --- | --- | --- |
| _Lights1ON | Yes | Sunrise1 OR Sunrise1sub |
| _Lights1OFF | Yes | NOT(Sunrise1) OR NOT(Sunrise1sub) |
| _Cooling1ON | Yes | (Lights1IsON AND TempDayA1) OR (NOT(Lights1IsON) AND TempNightA1) |
| _Cooling1OFF | Yes | (Lights1IsON AND TempDayB1) OR (NOT(Lights1IsON) AND TempNightB1) |
| _Humidity1ON | Yes | ( Cooling1IsON AND (Lights1IsON AND ( (Hum1Vs1IsON AND Hum1CB84) OR (Hum1Vs2IsON AND Hum1CB86) OR (Hum1Vs3IsON AND Hum1CB88) OR (Hum1Vs4IsON AND Hum1CB90) ) ) OR (NOT(Lights1IsON) AND ( (Hum1Vs1IsON AND Hum1CB76) OR (Hum1Vs2IsON AND Hum1CB78) OR (Hum1Vs3IsON AND Hum1CB80) OR (Hum1Vs4IsON AND Hum1CB82) ) ) ) OR ( NOT(Cooling1IsON) AND (Lights1IsON AND ( (Hum1Vs1IsON AND Hum1CB68) OR (Hum1Vs2IsON AND Hum1CB72) OR (Hum1Vs3IsON AND Hum1CB76) OR (Hum1Vs4IsON AND Hum1CB78) ) ) OR (NOT(Lights1IsON) AND ( (Hum1Vs1IsON AND Hum1CB61) OR (Hum1Vs2IsON AND Hum1CB65) OR (Hum1Vs3IsON AND Hum1CB68) OR (Hum1Vs4IsON AND Hum1CB72) ) ) ) |
| _Humidity1OFF | Yes | ( Cooling1IsON AND (Lights1IsON AND ( (Hum1Vs1IsON AND Hum1CA83) OR (Hum1Vs2IsON AND Hum1CA85) OR (Hum1Vs3IsON AND Hum1CA87) OR (Hum1Vs4IsON AND Hum1CA89) ) ) OR (NOT(Lights1IsON) AND ( (Hum1Vs1IsON AND Hum1CA75) OR (Hum1Vs2IsON AND Hum1CA77) OR (Hum1Vs3IsON AND Hum1CA79) OR (Hum1Vs4IsON AND Hum1CA81) ) ) ) OR ( NOT(Cooling1IsON) AND (Lights1IsON AND ( (Hum1Vs1IsON AND Hum1CA67) OR (Hum1Vs2IsON AND Hum1CA71) OR (Hum1Vs3IsON AND Hum1CA75) OR (Hum1Vs4IsON AND Hum1CA77) ) ) OR (NOT(Lights1IsON) AND ( (Hum1Vs1IsON AND Hum1CA60) OR (Hum1Vs2IsON AND Hum1CA64) OR (Hum1Vs3IsON AND Hum1CA67) OR (Hum1Vs4IsON AND Hum1CA71) ) ) ) |
| _Hum1Vs1ON | No | Hum1TestIsON AND Hum1Vs2IsON AND Hum1MA85 |
| _Hum1Vs2ON | No | Hum1TestIsON AND ( (Hum1Vs1IsON AND Hum1MB86) OR (Hum1Vs3IsON AND Hum1MA77) ) |
| _Hum1Vs3ON | No | ( Hum1TestIsON AND ( (Hum1Vs2IsON AND Hum1MB78) OR (Hum1Vs4IsON AND Hum1MA71) ) ) OR ( NOT(Hum1Vs1IsON) AND NOT(Hum1Vs2IsON) AND NOT(Hum1Vs3IsON) AND NOT(Hum1Vs4IsON) ) |
| _Hum1Vs4ON | No | Hum1TestIsON AND Hum1Vs3IsON AND Hum1MB72 |
| _Hum1Vs1OFF | No | _Hum1Vs2ON OR _Hum1Vs3ON OR _Hum1Vs4ON |
| _Hum1Vs2OFF | No | _Hum1Vs1ON OR _Hum1Vs3ON OR _Hum1Vs4ON |
| _Hum1Vs3OFF | No | _Hum1Vs1ON OR _Hum1Vs2ON OR _Hum1Vs4ON |
| _Hum1Vs4OFF | No | _Hum1Vs1ON OR _Hum1Vs2ON OR _Hum1Vs3ON |
| Hum1VsTrigger | Yes | Cooling1IsON; NOW>20 <50 |
| Hum1VsTrOFF | Yes | Hum1TestIsON; NOW>40 |

**Actions**

**Actions for Condition: _Cooling1OFF**

**Device Actions:**

**Immediate**

| **Device** | **Action** | **Arguments** |
| --- | --- | --- |
| Cooling1 | SetTarget | newTargetValue=0 |

**Actions for Condition: _Cooling1ON**

**Device Actions:**

**Immediate**

| **Device** | **Action** | **Arguments** |
| --- | --- | --- |
| Cooling1 | SetTarget | newTargetValue=1 |

**Actions for Condition: _Hum1Vs1OFF**

**Device Actions:**

**Immediate**

| **Device** | **Action** | **Arguments** |
| --- | --- | --- |
| Hum1Vs1 | SetTarget | newTargetValue=0 |

**Actions for Condition: _Hum1Vs1ON**

**Device Actions:**

**Immediate**

| **Device** | **Action** | **Arguments** |
| --- | --- | --- |
| Hum1Vs1 | SetTarget | newTargetValue=1 |

**Actions for Condition: _Hum1Vs2OFF**

**Device Actions:**

**Immediate**

| **Device** | **Action** | **Arguments** |
| --- | --- | --- |
| Hum1Vs2 | SetTarget | newTargetValue=0 |

**Actions for Condition: _Hum1Vs2ON**

**Device Actions:**

**Immediate**

| **Device** | **Action** | **Arguments** |
| --- | --- | --- |
| Hum1Vs2 | SetTarget | newTargetValue=1 |

**Actions for Condition: _Hum1Vs3OFF**

**Device Actions:**

**Immediate**

| **Device** | **Action** | **Arguments** |
| --- | --- | --- |
| Hum1Vs3 | SetTarget | newTargetValue=0 |

**Actions for Condition: _Hum1Vs3ON**

**Device Actions:**

**Immediate**

| **Device** | **Action** | **Arguments** |
| --- | --- | --- |
| Hum1Vs3 | SetTarget | newTargetValue=1 |

**Actions for Condition: _Hum1Vs4OFF**

**Device Actions:**

**Immediate**

| **Device** | **Action** | **Arguments** |
| --- | --- | --- |
| Hum1Vs4 | SetTarget | newTargetValue=0 |

**Actions for Condition: _Hum1Vs4ON**

**Device Actions:**

**Immediate**

| **Device** | **Action** | **Arguments** |
| --- | --- | --- |
| Hum1Vs4 | SetTarget | newTargetValue=1 |

**Actions for Condition: _Humidity1OFF**

**Device Actions:**

**Immediate**

| **Device** | **Action** | **Arguments** |
| --- | --- | --- |
| Humidity1 | SetTarget | newTargetValue=0 |

**Actions for Condition: _Humidity1ON**

**Device Actions:**

**Immediate**

| **Device** | **Action** | **Arguments** |
| --- | --- | --- |
| Humidity1 | SetTarget | newTargetValue=1 |

**Actions for Condition: _Lights1OFF**

**Device Actions:**

**Immediate**

| **Device** | **Action** | **Arguments** |
| --- | --- | --- |
| Lights1 | SetTarget | newTargetValue=0 |
| NightHeater1 | SetTarget | newTargetValue=1 |

**Actions for Condition: _Lights1ON**

**Device Actions:**

**Immediate**

| **Device** | **Action** | **Arguments** |
| --- | --- | --- |
| Lights1 | SetTarget | newTargetValue=1 |
| NightHeater1 | SetTarget | newTargetValue=0 |

**Actions for Condition: Hum1VsTrigger**

**Device Actions:**

**Immediate**

| **Device** | **Action** | **Arguments** |
| --- | --- | --- |
| Hum1Test | SetTarget | newTargetValue=1 |

**Actions for Condition: Hum1VsTrOFF**

**Device Actions:**

**Immediate**

| **Device** | **Action** | **Arguments** |
| --- | --- | --- |
| Hum1Test | SetTarget | newTargetValue=0 |

(2) The program for the second growth chamber. Note that the target temperature and RH are the same between the first and second growth chambers, but the parameter values needed to be slightly modified as two growth chambers are not exactly the same.

**Triggers**

| **Name** | **Description** |
| --- | --- |
| Cooling2IsON | Cooling2 is turned on |
| Hum2CA58 | Humidity reported by _Embedded Humidity Sensor go above 58 |
| Hum2CA62 | Humidity reported by _Embedded Humidity Sensor go above 62 |
| Hum2CA65 | Humidity reported by _Embedded Humidity Sensor go above 65 |
| Hum2CA69 | Humidity reported by _Embedded Humidity Sensor go above 69 |
| Hum2CA73 | Humidity reported by _Embedded Humidity Sensor go above 73 |
| Hum2CA75 | Humidity reported by _Embedded Humidity Sensor go above 75 |
| Hum2CA78 | Humidity reported by _Embedded Humidity Sensor go above 78 |
| Hum2CA79 | Humidity reported by _Embedded Humidity Sensor go above 79 |
| Hum2CA81 | Humidity reported by _Embedded Humidity Sensor go above 81 |
| Hum2CA82 | Humidity reported by _Embedded Humidity Sensor go above 82 |
| Hum2CA85 | Humidity reported by _Embedded Humidity Sensor go above 85 |
| Hum2CA89 | Humidity reported by _Embedded Humidity Sensor go above 89 |
| Hum2CB59 | Humidity reported by _Embedded Humidity Sensor go below 59 |
| Hum2CB63 | Humidity reported by _Embedded Humidity Sensor go below 63 |
| Hum2CB66 | Humidity reported by _Embedded Humidity Sensor go below 66 |
| Hum2CB70 | Humidity reported by _Embedded Humidity Sensor go below 70 |
| Hum2CB74 | Humidity reported by _Embedded Humidity Sensor go below 74 |
| Hum2CB76 | Humidity reported by _Embedded Humidity Sensor go below 76 |
| Hum2CB79 | Humidity reported by _Embedded Humidity Sensor go below 79 |
| Hum2CB80 | Humidity reported by _Embedded Humidity Sensor go below 80 |
| Hum2CB82 | Humidity reported by _Embedded Humidity Sensor go below 82 |
| Hum2CB83 | Humidity reported by _Embedded Humidity Sensor go below 83 |
| Hum2CB86 | Humidity reported by _Embedded Humidity Sensor go below 86 |
| Hum2CB90 | Humidity reported by _Embedded Humidity Sensor go below 90 |
| Hum2MA73 | Humidity reported by _Embedded Humidity Sensor go above 73 |
| Hum2MA78 | Humidity reported by _Embedded Humidity Sensor go above 78 |
| Hum2MA85 | Humidity reported by _Embedded Humidity Sensor go above 85 |
| Hum2MB74 | Humidity reported by _Embedded Humidity Sensor go below 74 |
| Hum2MB79 | Humidity reported by _Embedded Humidity Sensor go below 79 |
| Hum2MB86 | Humidity reported by _Embedded Humidity Sensor go below 86 |
| Hum2TestIsON | Hum2Test is turned on |
| Hum2Vs1IsON | Hum2Vs1 is turned on |
| Hum2Vs2IsON | Hum2Vs2 is turned on |
| Hum2Vs3IsON | Hum2Vs3 is turned on |
| Hum2Vs4IsON | Hum2Vs4 is turned on |
| Lights2IsON | Lights2 is turned on |
| Temp2MA73 | _Embedded Temperature Sensor temperature goes above 73 degrees |
| Temp2MB71 | _Embedded Temperature Sensor temperature goes below 71 degrees |
| TempDayA2 | _Embedded Temperature Sensor temperature goes above 67 degrees |
| TempDayB2 | _Embedded Temperature Sensor temperature goes below 68 degrees |
| TempNightA2 | _Embedded Temperature Sensor temperature goes above 71 degrees |
| TempNightB2 | _Embedded Temperature Sensor temperature goes below 72 degrees |

**Schedules**

| **Name** | **On Type** | **On Time** | **On Days** | **Random On Delay** | **Off After Type** | **Off Time** | **Off Days** | **Random Off Delay** |
| --- | --- | --- | --- | --- | --- | --- | --- | --- |
| Sunrise2 | Weekly | 11:00:00 | 1,2,3,4,5,6,7 | 30 | Weekly | 21:30:00 | 1,2,3,4,5,6,7 | 30 |
| Sunrise2sub | Weekly | 11:10:00 | 1,2,3,4,5,6,7 | 30 | Weekly | 21:40:00 | 1,2,3,4,5,6,7 | 30 |

**Conditions**

| **Name** | **Repeat** | **Expression** |
| --- | --- | --- |
| _Lights2ON | Yes | Sunrise2 OR Sunrise2sub |
| _Lights2OFF | Yes | NOT(Sunrise2) OR NOT(Sunrise2sub) |
| _Humidity2ON | Yes | ( Cooling2IsON AND (Lights2IsON AND ( (Hum2Vs1IsON AND Hum2CB80) OR (Hum2Vs2IsON AND Hum2CB83) OR (Hum2Vs3IsON AND Hum2CB86) OR (Hum2Vs4IsON AND Hum2CB90) ) ) OR (NOT(Lights2IsON) AND ( (Hum2Vs1IsON AND Hum2CB74) OR (Hum2Vs2IsON AND Hum2CB76) OR (Hum2Vs3IsON AND Hum2CB79) OR (Hum2Vs4IsON AND Hum2CB82) ) ) ) OR ( NOT(Cooling2IsON) AND (Lights2IsON AND ( (Hum2Vs1IsON AND Hum2CB66) OR (Hum2Vs2IsON AND Hum2CB70) OR (Hum2Vs3IsON AND Hum2CB76) OR (Hum2Vs4IsON AND Hum2CB79) ) ) OR (NOT(Lights2IsON) AND ( (Hum2Vs1IsON AND Hum2CB59) OR (Hum2Vs2IsON AND Hum2CB63) OR (Hum2Vs3IsON AND Hum2CB66) OR (Hum2Vs4IsON AND Hum2CB70) ) ) ) |
| _Humidity2OFF | Yes | ( Cooling2IsON AND (Lights2IsON AND ( (Hum2Vs1IsON AND Hum2CA79) OR (Hum2Vs2IsON AND Hum2CA82) OR (Hum2Vs3IsON AND Hum2CA85) OR (Hum2Vs4IsON AND Hum2CA89) ) ) OR (NOT(Lights2IsON) AND ( (Hum2Vs1IsON AND Hum2CA73) OR (Hum2Vs2IsON AND Hum2CA75) OR (Hum2Vs3IsON AND Hum2CA78) OR (Hum2Vs4IsON AND Hum2CA81) ) ) ) OR ( NOT(Cooling2IsON) AND (Lights2IsON AND ( (Hum2Vs1IsON AND Hum2CA65) OR (Hum2Vs2IsON AND Hum2CA69) OR (Hum2Vs3IsON AND Hum2CA75) OR (Hum2Vs4IsON AND Hum2CA78) ) ) OR (NOT(Lights2IsON) AND ( (Hum2Vs1IsON AND Hum2CA58) OR (Hum2Vs2IsON AND Hum2CA62) OR (Hum2Vs3IsON AND Hum2CA65) OR (Hum2Vs4IsON AND Hum2CA69) ) ) ) |
| _Cooling2ON | Yes | (Lights2IsON AND TempDayA2) OR (NOT(Lights2IsON) AND TempNightA2) |
| _Cooling2OFF | Yes | (Lights2IsON AND TempDayB2) OR (NOT(Lights2IsON) AND TempNightB2) |
| _Hum2Vs1ON | No | Hum2TestIsON AND Hum2Vs2IsON AND Hum2MA85 |
| _Hum2Vs2ON | No | Hum2TestIsON AND ( (Hum2Vs1IsON AND Hum2MB86) OR (Hum2Vs3IsON AND Hum2MA78) ) |
| _Hum2Vs3ON | No | ( Hum2TestIsON AND ( (Hum2Vs2IsON AND Hum2MB79) OR (Hum2Vs4IsON AND Hum2MA73) ) ) OR ( NOT(Hum2Vs1IsON) AND NOT(Hum2Vs2IsON) AND NOT(Hum2Vs3IsON) AND NOT(Hum2Vs4IsON) ) |
| _Hum2Vs4ON | No | Hum2TestIsON AND Hum2Vs3IsON AND Hum2MB74 |
| _Hum2Vs1OFF | No | _Hum2Vs2ON OR _Hum2Vs3ON OR _Hum2Vs4ON |
| _Hum2Vs2OFF | No | _Hum2Vs1ON OR _Hum2Vs3ON OR _Hum2Vs4ON |
| _Hum2Vs3OFF | No | _Hum2Vs1ON OR _Hum2Vs2ON OR _Hum2Vs4ON |
| _Hum2Vs4OFF | No | _Hum2Vs1ON OR _Hum2Vs2ON OR _Hum2Vs3ON |
| Hum2VsTrigger | Yes | Cooling2IsON; NOW>20 <50 |
| Hum2VsTrOFF | Yes | Hum2TestIsON; NOW>40 |

**Actions**

**Actions for Condition: _Cooling2OFF**

***Device Actions:***

**Immediate**

| **Device** | **Action** | **Arguments** |
| --- | --- | --- |
| Cooling2 | SetTarget | newTargetValue=0 |

**Actions for Condition: _Cooling2ON**

***Device Actions:***

**Immediate**

| **Device** | **Action** | **Arguments** |
| --- | --- | --- |
| Cooling2 | SetTarget | newTargetValue=1 |

**Actions for Condition: _Hum2Vs1OFF**

***Device Actions:***

**Immediate**

| **Device** | **Action** | **Arguments** |
| --- | --- | --- |
| Hum2Vs1 | SetTarget | newTargetValue=0 |

**Actions for Condition: _Hum2Vs1ON**

***Device Actions:***

**Immediate**

| **Device** | **Action** | **Arguments** |
| --- | --- | --- |
| Hum2Vs1 | SetTarget | newTargetValue=1 |

**Actions for Condition: _Hum2Vs2OFF**

***Device Actions:***

**Immediate**

| **Device** | **Action** | **Arguments** |
| --- | --- | --- |
| Hum2Vs2 | SetTarget | newTargetValue=0 |

**Actions for Condition: _Hum2Vs2ON**

***Device Actions:***

**Immediate**

| **Device** | **Action** | **Arguments** |
| --- | --- | --- |
| Hum2Vs2 | SetTarget | newTargetValue=1 |

**Actions for Condition: _Hum2Vs3OFF**

***Device Actions:***

**Immediate**

| **Device** | **Action** | **Arguments** |
| --- | --- | --- |
| Hum2Vs3 | SetTarget | newTargetValue=0 |

**Actions for Condition: _Hum2Vs3ON**

***Device Actions:***

**Immediate**

| **Device** | **Action** | **Arguments** |
| --- | --- | --- |
| Hum2Vs3 | SetTarget | newTargetValue=1 |

**Actions for Condition: _Hum2Vs4OFF**

***Device Actions:***

**Immediate**

| **Device** | **Action** | **Arguments** |
| --- | --- | --- |
| Hum2Vs4 | SetTarget | newTargetValue=0 |

**Actions for Condition: _Hum2Vs4ON**

***Device Actions:***

**Immediate**

| **Device** | **Action** | **Arguments** |
| --- | --- | --- |
| Hum2Vs4 | SetTarget | newTargetValue=1 |

**Actions for Condition: _Humidity2OFF**

***Device Actions:***

**Immediate**

| **Device** | **Action** | **Arguments** |
| --- | --- | --- |
| Humidity2 | SetTarget | newTargetValue=0 |

**Actions for Condition: _Humidity2ON**

***Device Actions:***

**Immediate**

| **Device** | **Action** | **Arguments** |
| --- | --- | --- |
| Humidity2 | SetTarget | newTargetValue=1 |

**Actions for Condition: _Lights2OFF**

***Device Actions:***

**Immediate**

| **Device** | **Action** | **Arguments** |
| --- | --- | --- |
| Lights2 | SetTarget | newTargetValue=0 |
| NightHeater2 | SetTarget | newTargetValue=1 |

**Actions for Condition: _Lights2ON**

***Device Actions:***

**Immediate**

| **Device** | **Action** | **Arguments** |
| --- | --- | --- |
| Lights2 | SetTarget | newTargetValue=1 |
| NightHeater2 | SetTarget | newTargetValue=0 |

**Actions for Condition: Hum2VsTrigger**

***Device Actions:***

**Immediate**

| **Device** | **Action** | **Arguments** |
| --- | --- | --- |
| Hum2Test | SetTarget | newTargetValue=1 |

**Actions for Condition: Hum2VsTrOFF**

***Device Actions:***

**Immediate**

| **Device** | **Action** | **Arguments** |
| --- | --- | --- |
| Hum2Test | SetTarget | newTargetValue=0 |
